# The Spinnaker-Sail Sign: Neonatal Pneumomediastinum

**DOI:** 10.5334/jbsr.1589

**Published:** 2018-08-07

**Authors:** Simon Vanden Berghe, F. Devlies, P. Seynaeve

**Affiliations:** 1 KU Leuven, BE; 2 AZ Groeninge, BE

**Keywords:** Neonate, pneumomediastinum, spinnaker, sail, sign

## Case Report

A 34-year-old woman has a vaginal partus after 38 weeks and one day of pregnancy. The delivery is induced because of pregnancy diabetes. There are no complications during delivery with epidural anesthesia. The neonate is a boy with a weight of 3150 g and a length of 50 cm. APGAR scores are 8 and 9 after one and five minutes. Twenty-five minutes after delivery the neonate begins to grunt and shows tachypnea. Saturation levels remain good at 96% without the need of extra oxygenation. There are no signs of cardiocirculatory distress.

**Figure 1:**
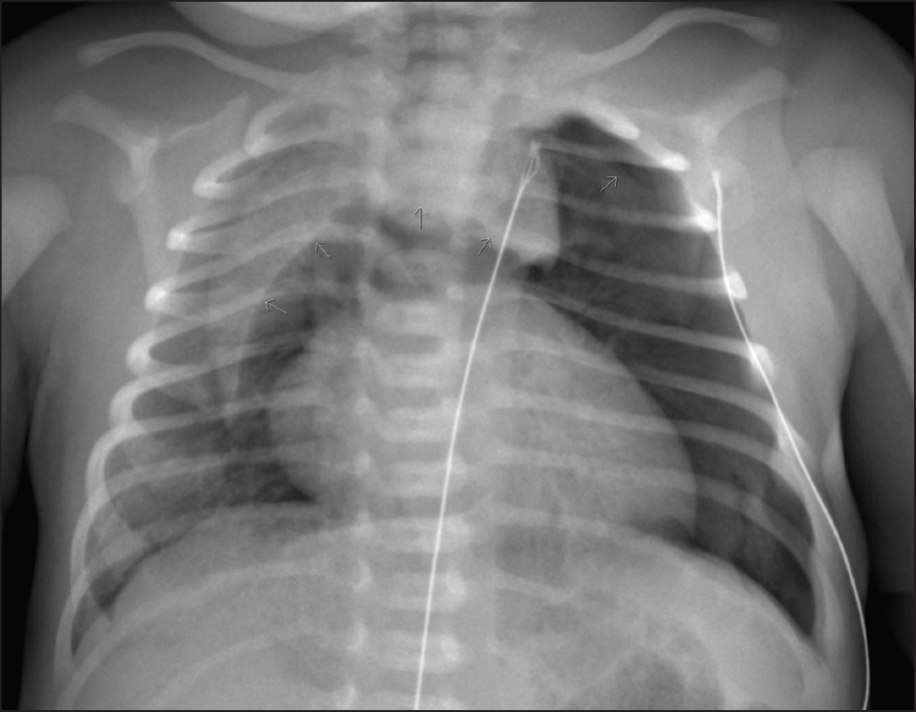
Frontal plain radiograph of the chest, showing the Spinnaker-Sail Sign. Both lobes of the thymus are lifted and displaced into the upper mediastinum, creating a wedge-shaped opacity. This resembles a Spinnaker sail. A pneumothorax can be seen at the left lung apex.

**Figure 2:**
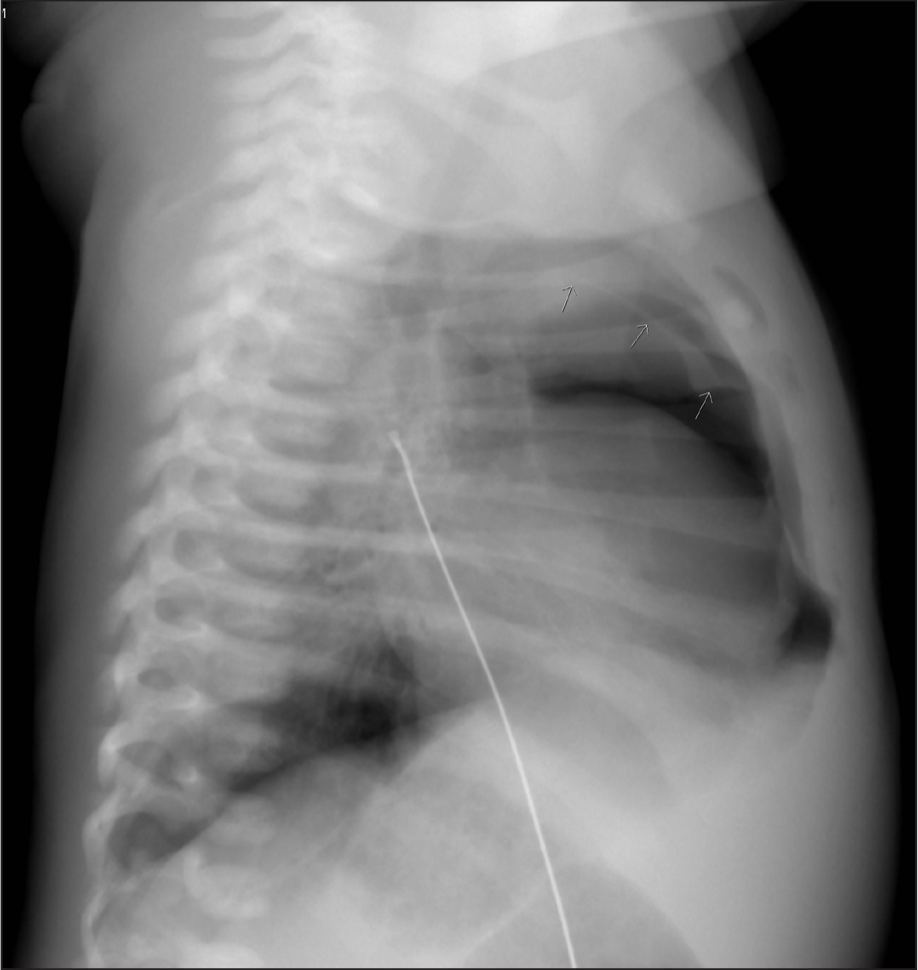
Lateral plain radiograph of the chest. The mediastinal air can be seen as a translucency in the areas surrounding the heart with a sharp delineation.

A chest X-ray (Figures 1 and 2) shows a pneumothorax at the left lung apex and the Spinnaker-Sail sign, a sign of pneumomediastinum. Because of the favorable cardiocirculatory condition of the neonate and the minor need for oxygenation, the clinicians opt for a conservative approach. The patient is admitted in the neonatal care unit and receives extra oxygenation in an incubator.

Oxygenation is decreased during the following days and is ceased on day three. There is a favorable clinical evolution, with minor tachypnea and desaturation during breastfeeding. On day eight the respiratory function is back to normal.

## Discussion

The Spinnaker-Sail sign can be seen on a frontal plain radiograph of the chest and is characteristic for a pneumomediastinum in neonates. Both lobes of the thymus are lifted and displaced laterally due to the air in the mediastinum. This creates a wedge-shaped opacity extending into the superior mediastinum that has a sharp delineation inferiorly by the translucent mediastinal air. The Spinnaker-Sail sign is named after the headsail of a boat, which has a similar shape when it is stretched by the wind [1]. Another name for this sign is the ‘Angel Wing’ sign.

Pneumomediastinum occurs in approximately 1 per 1000 births. Sometimes the pneumomediastinum can occur due to positive pressure ventilation, birth trauma or meconium aspiration, although it is often idiopathic [1]. In idiopathic pneumomediastinum, a difference in pressure between the alveoli and the surrounding tissues causes alveolar rupture. This allows air to escape alongside the peribronchial and perivascular structures to the mediastinum. In most cases the infant is asympomatic and not every case is detected. When the neonate remains stable, a conservative approach is the treatment of choice and a control chest radiograph can be used for follow-up [1].
